# Circular polarization sensitive absorbers based on graphene

**DOI:** 10.1038/srep23897

**Published:** 2016-04-01

**Authors:** Kunpeng Yang, Min Wang, Mingbo Pu, Xiaoyu Wu, Hui Gao, Chenggang Hu, Xiangang Luo

**Affiliations:** 1 State Key Laboratory of Optical Technologies on Nano-Fabrication and Micro-Engineering, Institute of Optics and Electronics, Chinese Academy of Science, P.O. Box 350, Chengdu 610209, China

## Abstract

It is well known that the polarization of a linearly polarized (LP) light would rotate after passing through a single layer graphene under the bias of a perpendicular magnetostatic field. Here we show that a corresponding phase shift could be expected for circularly polarized (CP) light, which can be engineered to design the circular polarization sensitive devices. We theoretically validate that an ultrathin graphene-based absorber with the thickness about λ/76 can be obtained, which shows efficient absorption >90% within incident angles of ±80°. The angle-independent phase shift produced by the graphene is responsible for the nearly omnidirectional absorber. Furthermore, a broadband absorber in frequencies ranging from 2.343 to 5.885 THz with absorption over 90% is designed by engineering the dispersion of graphene.

Graphene-based absorbers have attracted immense research attentions because of the advantages of ultrathin profile, highly thermal transport and fast external excitation-response^1^,^2^,^3^,^4^,^5^,^6^. When resorting to the localized surface plasmon polariton (LSPP) resonance^1^, the surface plasmon polariton (SPP) transmission loss^2^ or the cavity resonance^3^,^4^, efficient absorption has been obtained in many graphene-based structures. Graphene is the mono-atomic carbon material described by the two-dimensional (2D) Dirac-like equation^7^. Owing to the gapless energy band^8^,^9^, graphene shows unique properties that do not exist in conventional conducting materials, such as quantum Hall effect^10^,^11^, giant Faraday rotation^12^,^13^ and Kerr effect^14^,^15^, leading to many photo- and magneto-electric applications^16^,^17^,^18^. Faraday rotation, the polarization rotation of the plane wave when passing through the transparent medium in the perpendicular magnetostatic field, is an important phenomenon of the magneto-optic effect of the linearly polarized (LP) light, which naturally originates from the circular motion of the conducting electrons in the 2D graphene sheet. As known to all, the circularly polarized (CP) light can be decomposed into two orthotropic LP light. When the graphene sheet is illuminated by a CP light at normal direction and biased by a perpendicular magnetostatic field, a phase shift is expected across the graphene, which bears similar essence as the geometric phase in metasurface^19^,^20^. By reasonably engineering the phase shift, kinds of the magneto-controlled devices can be designed, including the circular polarization sensitive absorbers.

For the traditional sandwich-type absorbers, suitable impedance match between input impedance and air impedance and phase difference between incident and reflected waves are necessary to achieve perfect absorption. In the following, the phase difference is mainly considered while impedance match can be achieved easily by doping or loading the resistive elements^21^,^22^,^23^,^24^. In the graphene-based absorber, the total phase difference *φ*_*total*_ = 2*φ*_*screen*_ + 2*φ*_*d*′_ + *π* between incident and reflected light contains three parts: the phase shift caused by the screen *φ*_*screen*_, the propagation phase delay in the dielectric space *φ*_*d*′_ (*d*′ is the relative thickness of the graphene-based absorber) and *π* phase shift of the metallic mirror. The sign of *φ*_*screen*_ is decided by the capacitive or inductive features of the screen, which plays a crucial role in guaranteeing the efficient absorption.

In this work, we analyze the effect of the phase shift produced by the graphene screen in designing various CP light absorbers. The advantages of the absorber rely on the magnetic circular dichroism and the adjustable absorption property by changing the magnetostatic bias or the chemical potential (Fermi energy). To realize the omnidirectional absorption, the phase shift *φ*_*screen*_ should be the dominant component of the total phase difference. The simulation results show that the left-handed circularly polarized (LCP) incident light within the angle range of ±80° is efficiently absorbed by the graphene structure with a thickness of about *λ*/76 (*λ* is the wavelength of the incident light), while almost all the right-handed circularly polarized (RCP) incident light is reflected. The absorption mechanism is different from the cavity resonance in the nanovoids^25^,^26^ and the magnetic coupling in the metamaterial absorbers^27^,^28^,^29^. Moreover, a broadband absorber can be designed using the phase shift to broaden the operation frequency range. The absorption is above 90% in the region of 2.343 to 5.885 THz.

## Results

### Phase shift of the grapheme

The conductivity is usually adopted to describe the electromagnetic characteristic of the 2D graphene sheet. In the low frequency region, the interband transition is dominant in the electron motion of the graphene and the conductivity is well approximated by the Drude model^30^. Under the control of the magnetostatic bias *B*, the conductivity is anisotropic with items in the off-axis diagonal^31^,^32^. The tensor expression for the linear polarization is [*σ*] = (*σ*_*xx*_, *σ*_*xy*_; *σ*_*yx*_, *σ*_*yy*_) with


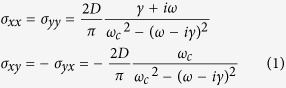


where *D* is the Drude weight, *γ* is the scattering rate, 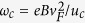 is the cyclotron frequency, *u*_*c*_ is the chemical potential, *v*_*F*_ is the Fermi velocity and *e* is the electron charge. For the CP incident light, the conductivity of the graphene is calculated as^33^:


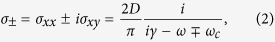


where the signs ‘+’ and ‘−’ stand for RCP and LCP light, respectively. Furthermore, surface impedances can be accurately written as:


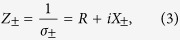


which can be considered as the superposition of the polarization-independent resistance *R* = *πγ*/2*D* and the frequency-dependent inductive component *X*_± _ = *π*(*ω* ± *ω*_*c*_)/2*D*.

In the following, we consider the transmission at the interface of the graphene and the semi-infinite non-magnetic dielectric space with a refractive index of *n*. According to the metasurface-assisted Fresnel’s equation (MAFE)^34^, the phase shift can be calculated as:


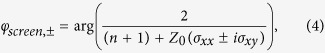


where *Z*_*0*_ is the impedance of the uniform graphene for *B* = 0 Tesla. Clearly, the phase shifts for RCP and LCP light are different, which could be combined to obtain the Faraday rotation angle:


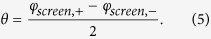


which is different but similar to the expression given in previous article^12^.

**Figure 1:**
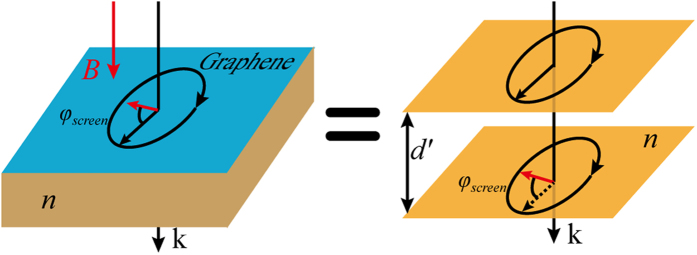
Phase shift in the perpendicular magnetostatic field.

A shown in Fig. 1, the phase shift induced by the graphene sheet can be regarded to be equivalent to the accumulated phase in the propagation of light with a distance of *d*′ = *φ*_*screen*_/*kn* in the dielectric space. Obviously, the phase shift is intrinsically related to the relative thickness of the absorber.

### Ultrathin and omnidirectional absorber

Designing an efficient and omnidirectional absorber with an ultrathin profile with traditional approaches is challenging, since the electromagnetic coupling and the resonance usually become much weaker for smaller mode area. The absorbers based on the magnetic resonance are nearly angle-independent^29^. However, further reduction of the thickness seems to be not a trivial thing. Here we show that it is possible to construct an ultrathin and omnidirectional absorber using the phase shift produced by the graphene sheet.

In principle, to obtain perfect absorption, the resistance *R* of the graphene must be equal to the real part of the complex impedance of the screen for the ideal absorber *Z*_*ideal*_ = *Z*_*0*_/(1 + *incot*(*knd*)) (*k* is the wave vector and *d* is the thickness of the space)^18^. Hence, the relative thickness *d*′ of the graphene-based absorber can be expressed as:


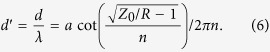


The minimal value of *R* is expected for achieving the ultrathin absorber. Since *R* is reduced as 1/*γ* or *u*_*c*_ increases, the minimal value of *γ* and the maximal value of *u*_*c*_ are required. In this case, *γ* = 0.2 meV is adopted from the theoretical estimation^35^ and *u*_*c*_ is set as 1 eV. The minimal value of *R* is obtained as 2.57 Ω per square and *d*′ is about *λ*/76. Then, phase match is considered. For an absorber with the dielectric space of *n* = 1.45 and the given thickness *d*′ = *λ*/76, the phase components are calculated as the propagation phase *φ*_*d*′_ = *knd*′ = 0.038*π* and the phase shift *φ*_*screen*_ = arg(Im(*t*)/Re(*t*)) = −0.4114*π*, where *t* is the transmission of the LCP light passing through the ideal screen covering on the semi-infinite dielectric material (*φ*_*screen*_ = 0.4102*π* for the RCP light). The negative phase shift means that only the LCP incident light can be absorbed. Since the phase shift is relative to the direction of the magnetic field, the absorption behaviors for the two incident light are reciprocal by changing the direction of the magnetic field.

**Figure 2:**
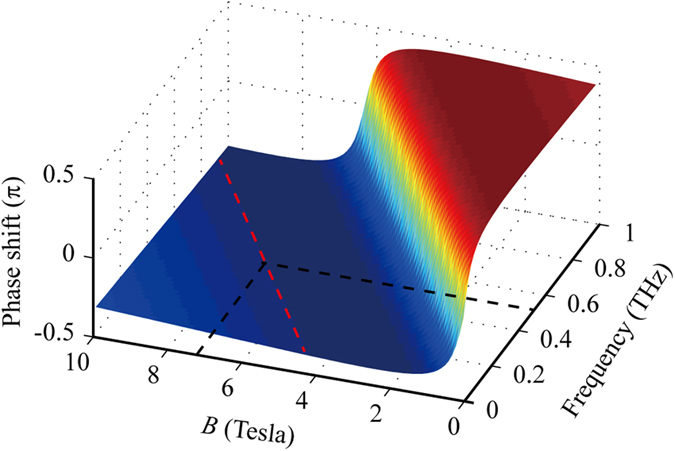
Phase shift *φ*_*screen*,−_ as the function of frequency and magnetic field.

Subsequently, the negative phase shift is utilized to demonstrate that the LCP light absorber can be achieved. The key point is whether the phase shift can be realized by the graphene under the magnetostatic bias. Figure 2 depicts the phase shift as the function of the frequency within 0~1 THz and the magnetostatic bias in the range of 0~10 Tesla. Clearly, arbitrary values within ±0.4189*π*, including the desired value of −0.4114*π*, can be obtained by tuning the bias. The magnetic bias has an almost linear relationship with the frequency in the given phase shift range, which can be derived as:


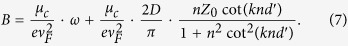


For the given parameters and the nonzero thickness, the second term is a constant. Taking the resonant frequency of 0.55 THz for instance, the desired magnetic field and the thickness of the space are *B* = 7.12 Tesla and *d*′ = 7.146 μm, respectively.

**Figure 3:**
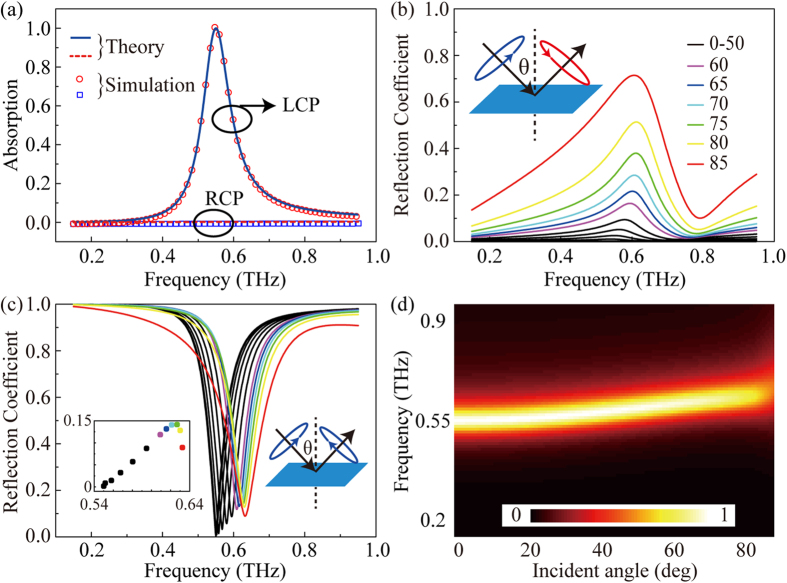
Omnidirectional absorption of the absorber. (**a**) Theory and simulation absorption of the absorber. (**b**) Reflection coefficients for the LCP incident light to the RCP reflected light under different incident angles. (**c**) Reflection coefficients for the LCP incident light to the LCP reflected light under different incident angles. The inset is the minimal reflection coefficients for different incident angles in details. (**d**) Absorption of the LCP incident light under different incident angles.

To validate the above conclusion, theory results are obtained using a transfer matrix method (TMM) coded in MATLAB software. As illustrated in Fig. 3a, the theory results show that the perfect absorption of the LCP light appears at 0.55 THz as expected. The absorption bandwidth is about 5.53% with the absorption over 90%. However, the RCP light is mostly reflected within 0.1~0.9 THz. The different absorption around 0.55 THz for LCP and RCP light indicate that the absorber is circular polarization-sensitive. Furthermore, the model of the graphene-based absorber is constructed using the commercial electromagnetic simulation software CST 2013. The simulation results shown in Fig. 3a agree well with the theory results. Significantly, we note that if the resonant frequency becomes lower, the required magnetic field will be reduced as illustrated in Fig. 2.

The angle-dependence of the ultrathin graphene-based absorber is also investigated. Propagation direction and electric field rotational direction of the LCP light are assumed to obey the left-handed rule. The LCP light would be absorbed or reflected as LCP or RCP light when illuminating the absorber. As presented in Fig. 3b, the reflection coefficient *r*_*1*_ describes the efficiency of the incident LCP light converted to RCP light for variable angles. Clearly, the RCP light could be hardly found within ±60° and a peak and a dip respectively appear at 0.6 THz and 0.8 THz as the incident angle increases. Figure 3c shows the coefficient *r*_*2*_ of the LCP reflected light for different incident angles and the inset gives the minimal values of the reflection coefficients in details. The maximum value is only about 0.15 when the angle is 75°. The results validate that the absorber would strongly suppress the transformation from the LCP incident light to the LCP reflected light around 0.55 THz. Considering the metal layer at the backside of the substrate, the transmission is zero and the absorption is calculated as 
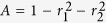
, as shown in Fig. 3d. Although there is a small blue shift of the resonant frequency as the incident angle increases, the absorber still exhibits rather high absorption. The inner mechanism is originated from the interference of waves between the graphene layer and the metal mirror, which is different from the cavity resonance in the nanovoids and the magnetic coupling in the metamaterial absorbers^26^,^29^. Moreover, the phase shift caused by the screen, rather than the propagation phase delay in the dielectric space, is dominant for the resonant frequency. The ratio *φ*_*screen*_/(*φ*_*screen*_ + *φ*_*d*′_) is about 91.5% at the normal incidence. The thicknesses of the graphene and the dielectric spacer are much smaller than the incident wavelength and the total phase difference changes little for different incident angles, leading to excellent wide-angle absorption property.

### Broadband CP light absorber

**Figure 4:**
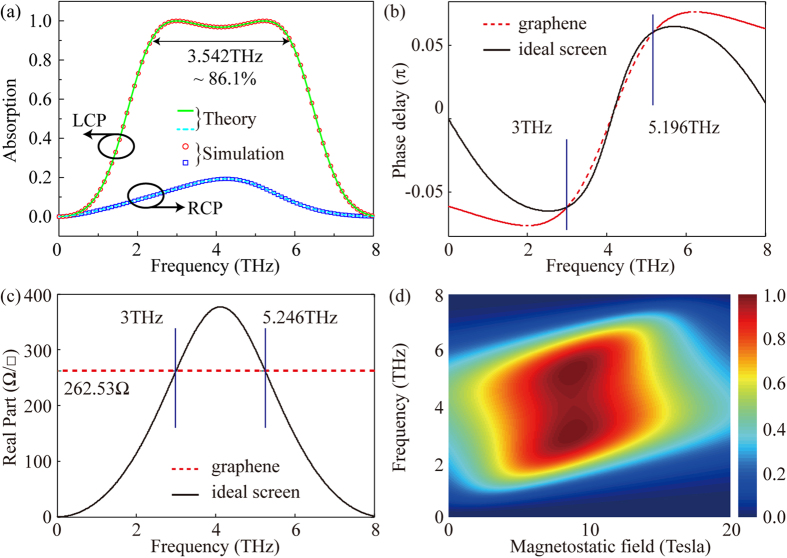
Broadband absorption of the absorber. (**a**) Theory and simulation absorption. (**b**) Phase shift and (**c**) resistance of the graphene (dashed line) and the ideal screen (solid line). (**d**) Absorption of the LCP light as the function of the magnetostatic bias and the frequency.

As shown in Eq. 3, the graphene sheet exhibits the inductive or capacitive dispersion characteristics at the frequency range below or beyond *ω*_*c*_ for the CP incident light. Thus, a dual-frequency resonance of the graphene is feasible to broaden the operation region. Different from the previous ultrathin absorber, the corresponding parameters are optimized as *u*_*c*_ = 0.34 eV and *γ* = 0.7 meV^36^. The resistance *R* is about 262.5 Ω per square and the relative thickness *d*′ is about *λ*/7.96, which is close to *λ*/4*n*. By setting the resonant frequency to be 3 THz, the desired magnetic field is calculated as 8.78 Tesla. Figure 4a is the theory and simulation results, which show that the absorption *A* for the LCP light is larger than 90% within 2.343 to 5.885 THz, while the RCP light is mostly reflected in the range of 0 to 8 THz. As illustrated in Fig. 4b, the red dashed line is the phase shift caused the graphene while the black line is the desired phase shift produced by the ideal screen for the perfect absorption^29^. Three intersections indicate that good phase matches are achieved at 3 THz, 4.1 THz and 5.196 THz. Considering the resistance match presented in Fig. 4c, two intersections at 3 THz and 5.196 THz are realized with nearly perfect absorption. Figure 4d shows the absorption is the function of the frequency and the magnetostatic bias. Obviously, the broadband absorption can be tuned by the magnetostatic bias.

## Discussion

**Figure 5:**
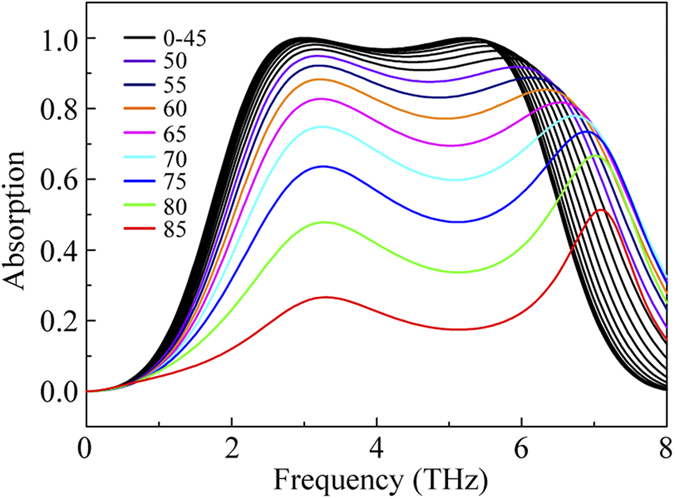
Simulation results of the broadband absorber under different incident angles.

Based the above results, the angle-dependence of the broadband LCP light absorber is also simulated, as presented in Fig. 5. The two resonant frequency points will shift as the incident angle increases. The absorption *A* is larger than 90% in the range of 0–45°, while the absorption will rapidly decrease due to the impedance mismatch when the incident angle is out of the range of 45°. We note that the absorption property of the broadband absorber is similar to the Salisbury screen.

In summary, we investigated the effect of the phase shift of the graphene originated from the Faraday rotation in designing the CP light sensitive absorbers under the perpendicular magnetostatic field. An ultrathin and omnidirectional absorber with a thickness about *λ*/76 can be realized when the phase shift induced by the graphene sheet is the dominant component of the total phase difference. Moreover, considering the inductive or capacitive characteristics of the graphene at the two sides of the cyclotron frequency, a broadband circular polarization sensitive absorber is obtained by inducing the dual-frequency resonance. The phase shift is also relative to the direction of the magnetic field, thus the absorption behaviors of LCP and RCP light are reciprocal by changing the direction of the magnetic field.

## Method

The theory results are obtained using the transfer matrix method (TMM) coding in MATLAB software, while the simulation results are achieved using the commercial electromagnetic simulation software CST 2013 based on the Finite Element Method (FEM). In the simulation, the graphene is considered as the thin layer with a thickness of *δ* = 10 nm. The ‘electric gyrotropic’ is described as the dispersion model of the thin graphene layer. The plasmon frequency 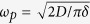 is independent on the magnetic field. The collision frequency and cyclotron frequency are 2π*γ* and *w*_*c*_, respectively.

## Additional Information

**How to cite this article**: Yang, K. *et al*. Circular polarization sensitive absorbers based on graphene. *Sci. Rep*. **6**, 23897; doi: 10.1038/srep23897 (2016).
